# Supplementary material for: Long-tailed distributions of inter-event times as mixtures of exponential distributions
Source: R Soc Open Sci. 2020 Feb 26;7(2):191643. doi: 10.1098/rsos.191643 (PMC7062064; doi:10.1098/rsos.191643)
Supplement: Supplementary Materials for “Long-tailed distributions of inter-event times as mixtures of exponential distributions” [file rsos191643supp1.pdf]

Supplementary Materials for "Long-tailed distributions of inter-event times as mixtures of exponential distributions"

Makoto Okada[1], Kenji Yamanishi[1], and Naoki Masuda[2,3,4*]

[1]Graduate School of Information Science and Technology, The University of Tokyo
[2]Department of Engineering Mathematics, University of Bristol, Clifton, Bristol, United Kingdom
[3]Department of Mathematics University at Buffalo, State University of New York, Buffalo, NY 14260-2900, USA
[4]Computational and Data-Enabled Science and Engineering Program, University at Buffalo, State University of New York, Buffalo, NY 14260-5030, USA
[*]Correspondence to naokimas@buffalo.edu

S1 Derivation of $\ell(m_{\min})$ and $\ell(m_{\max})$

The codelength for a natural number m, denoted by $\log^* m$, is given by[1]

$$\log^* m = \log c + \log m + \log\log m + \cdots . \tag{1}$$

In Equation (1), c is a constant approximately equal to 2.865 and is the largest lower bound for which the codelength for natural numbers satisfies the Kraft's inequality. The summation on the right-hand side of Equation (1) is considered up to the terms for which $\log\cdots\log m$ remains positive.

Because $m_{\min}$ or $m_{\max}$ may be negative, we add one bit to $\log^* m_{\min}$ and $\log^* m_{\max}$ to encode the sign of $m_{\min}$ and $m_{\max}$. Furthermore, because $m_{\min}$ or $m_{\max}$ may be equal to zero, for the sake of encoding, we shift any nonnegative value of $m_{\min}$ or $m_{\max}$ by $+1$ before the encoding. Therefore, we obtain

$$\ell(m_{\min}) = \log^*(|m_{\min}|+1) + \log 2 \tag{2}$$

and similarly for $\ell(m_{\max})$.

S2 Parameter values of the selected EMMs

For the individuals with the largest number of inter-event times in each data set, we compared EMMs having different numbers of initial components, k, using each of the six model selection criteria. For each data set, the EMMs selected under at least one criterion are compared with the empirical distribution in Figure 1. The parameter values obtained for the EMMs selected under at least one criterion are as follows.

For the Office data set, the model selection yielded $(k,k^*) = (2,2)$ with $\{(\hat{\pi}_j,\hat{\mu}_j)\}_{j=1}^{2} = \{(0.811, 1.398\times 10^2), (0.189, 2.391\times 10^3)\}$ under all the six criteria.

For the Hypertext data set, the model selection yielded $(k,k^*) = (2,2)$ with $\{(\hat{\pi}_j,\hat{\mu}_j)\}_{j=1}^{2} = \{(0.903, 6.955\times 10^1), (0.097, 1.290\times 10^3)\}$ for all the six criteria.

For the Reality data set, the model selection yielded $\{(\hat{\pi}_j,\hat{\mu}_j)\}_{j=1}^{2} = \{(0.957, 1.420\times 10^{-1}), (0.043, 2.030\times 10^0)\}$ when $(k,k^*) = (2,2)$ was selected, and $\{(\hat{\pi}_j,\hat{\mu}_j)\}_{j=1}^{3} = \{(0.848, 9.387\times 10^{-2}), (0.144, 6.647\times 10^{-1}), (0.008, 6.431\times 10^0)\}$ when $(k,k^*) = (3,3)$ was selected.

For the Bitcoin data set, the model selection yielded $\{(\hat{\pi}_j,\hat{\mu}_j)\}_{j=1}^{3} = \{(0.079, 2.160\times 10^2), (0.874, 2.026\times 10^5), (0.046, 1.924\times 10^6)\}$ when $(k,k^*) = (3,3)$ was selected, $\{(\hat{\pi}_j,\hat{\mu}_j)\}_{j=1}^{3} = \{(0.081, 2.351\times 10^2), (0.884, 2.135\times 10^5), (0.035, 2.231\times 10^6)\}$ when $(k,k^*) = (4,3)$ was selected, and $\{(\hat{\pi}_j,\hat{\mu}_j)\}_{j=1}^{4} = \{(0.079, 2.160\times 10^2), (0.012, 1.445\times 10^3), (0.868, 2.096\times 10^5), (0.041, 2.040\times 10^6)\}$ when $(k,k^*) = (5,4)$ was selected. In the second and third cases, we only reported three pairs $(\hat{\pi}_j,\hat{\mu}_j)$ because the other exponential distributions were judged to be unused by any individual.

For the Email data set, the model selection yielded $\{(\hat{\pi}_j,\hat{\mu}_j)\}_{j=1}^{4} = \{(0.020, 2.291\times 10^0), (0.690, 7.028\times 10^2), (0.274, 2.350\times 10^4), (0.016, 6.581\times 10^5)\}$ when $(k,k^*) = (4,4)$ was selected, and $\{(\hat{\pi}_j,\hat{\mu}_j)\}_{j=1}^{6} = \{(0.015, 1.683\times$

$10^0), (0.509, 3.129 \times 10^2), (0.321, 3.502 \times 10^3), (0.137, 3.940 \times 10^4), (0.017, 2.359 \times 10^5), (0.001, 8.952 \times 10^6)\}$ when $(k, k^*) = (8, 6)$ was selected.

For the College data set, the model selection yielded $\{(\hat{\pi}_j, \hat{\mu}_j)\}_{j=1}^3 = \{(0.709, 1.126 \times 10^2), (0.236, 1.299 \times 10^4), (0.055, 2.079 \times 10^5)\}$ when $(k, k^*) = (3, 3)$ was selected, $\{(\hat{\pi}_j, \hat{\mu}_j)\}_{j=1}^4 = \{(0.485, 3.997 \times 10^1), (0.261, 3.632 \times 10^2), (0.230, 2.320 \times 10^4), (0.024, 3.829 \times 10^5)\}$ when $(k, k^*) = (4, 4)$ was selected, and $\{(\hat{\pi}_j, \hat{\mu}_j)\}_{j=1}^6 = \{(0.408, 3.010 \times 10^1), (0.327, 2.706 \times 10^2), (0.137, 6.038 \times 10^3), (0.110, 4.710 \times 10^4), (0.002, 1.422 \times 10^5), (0.017, 4.976 \times 10^5)\}$ when $(k, k^*) = (8, 6)$ was selected.

For the Sexual data set, the model selection yielded $k^* = 1$ and $\{(\hat{\pi}_j, \hat{\mu}_j)\}_{j=1}^1 = \{(1.000, 1.063 \times 10^1)\}$ for each of $k = 1, 2$ and 3. In Figure 1, the corresponding distribution is labeled $(k, k^*) = (1, 1)$.

S3 Comparison of EMMs and Pareto distributions in terms of the AIC and BIC

Although neither the EMM nor the Pareto distribution allows the use of the AIC or BIC due to the lack of asymptotic normality of the maximum likelihood estimator, we compared the EMMs and Pareto distributions in terms of the AIC and BIC as a reference. The results are shown in Table S1. The results are qualitatively the same as those in Table 3, where the different models are compared in terms of the likelihood.

Table S1. AIC and BIC values for the maximum likelihood estimators compared between the EMM and Pareto distribution. We used the individuals that had the largest number of inter-event times in each data set, as we did so for Table 3 For the EMM, we used the (k, k^*) values that minimize the AIC, which are shown in Table 2.

	AIC		BIC	
Data	EMM	Pareto	EMM	Pareto
Office	2781.79	2806.40	2787.78	2810.40
Hypertext	3827.57	3536.81	3834.31	3541.31
Reality	-857.69	-2061.39	-844.97	-2056.30
Bitcoin	7971.99	8521.72	7991.81	8526.12
Email	36021.81	38855.35	36068.92	38861.63
College	8612.58	9075.08	8650.03	9080.08
Sexual	397.88	425.40	399.27	428.18

S4 Analysis of the Office and Hypertext data sets without excluding inter-event times across different days

In the main text, we analyzed the Office and Hypertext data sets after excluding the inter-event times that span multiple days. In this section, we include these long inter-event times and fit the EMMs. For the individuals with the largest number of inter-event times in each data set, we compared the likelihood for the EMM, Pareto distribution, and the PLFit depending on whether or not we exclude short inter-event times. The results are shown in Table S2. The results are qualitatively the same as those shown in the main text (Table 3).

The model selection for the EMMs yielded $(k, k^*) = (3, 3)$ for both data sets. These k and k^* values are larger by one than when the inter-event times spanning multiple days are removed (Table 2). This is because long inter-event times spanning multiple days contributed an exponential distribution to the EMMs. In fact, the estimated parameter values for the EMMs with $(k, k^*) = (3, 3)$ are $\{(\hat{\pi}_j, \hat{\mu}_j)\}_{j=1}^3 = \{(0.796, 1.398 \times 10^2), (0.185, 2.391 \times 10^3), (0.019, 9.364 \times 10^4)\}$ for the Office data set and $\{(\hat{\pi}_j, \hat{\mu}_j)\}_{j=1}^3 = \{(0.900, 6.955 \times 10^1), (0.097, 1.290 \times 10^3), (0.003, 4.420 \times 10^4)\}$ for the Hypertext data set. For both data sets, the two exponential distributions with the smallest means are indistinguishable from those when the EMMs are fitted to the data excluding multi-day inter-event times, i.e., $\{(\hat{\pi}_j, \hat{\mu}_j)\}_{j=1}^2 = \{(0.811, 1.398 \times 10^2), (0.189, 2.391 \times 10^3)\}$ for the Office data set and $\{(\hat{\pi}_j, \hat{\mu}_j)\}_{j=1}^2 = \{(0.903, 6.955 \times 10^1), (0.097, 1.290 \times 10^3)\}$ for the Hypertext data set.

The distribution of the selected number of components of the EMM across the individuals is shown in Figure S1 for each data set and model selection criterion. A comparison of Figures 2 and S1 confirms that the long inter-event times across days increase the number of components by one in most cases.

References

1. J. Rissanen. Stochastic complexity. J. R. Stat. Soc. Ser. B, 49:223–239, 1987.

Table S2. Likelihood of the entire and truncated data sets for the Office and Hypertext data sets when inter-event times spanning multiple days are retained. The results for the individual with the largest number of inter-event times in each data set are shown. For the EMM, we used $(k, k^*) = (3, 3)$, which minimized all the six criteria. See the caption of Table 3 for legends.

	all			$\tau > \min \tau_i$			$\tau \geq \hat{b}$			
Data	n	EMM	Pareto	n''	EMM	Pareto	n'	EMM	Pareto	PLFit
Office	411	−2918.7	−2930.5	395(96%)	−2834.0	−2869.3	355(86%)	−2616.5	−2676.3	−2477.5
Hypertext	661	−3863.0	−3569.4	466(70%)	−2960.7	−2947.7	339(51%)	−2336.8	−2382.2	−2069.3

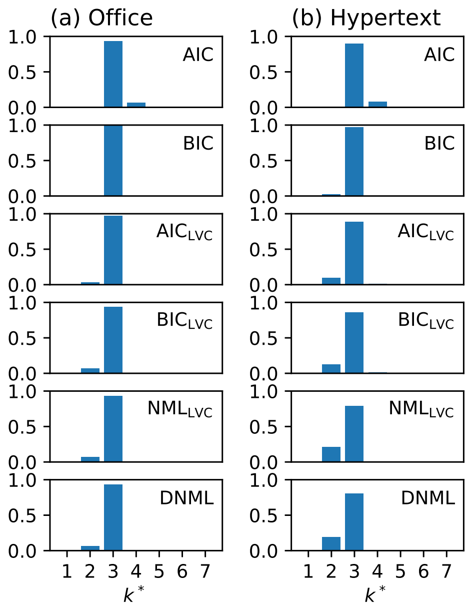

Figure S1. Distributions of the effective number of components in the EMM, k^*, when the inter-event times spanning multiple days are retained. (a) Office. (b) Hypertext. We calculated the distributions on the basis of the same individuals as those used in Figure 2.
